# Is use of CBCT without proper training justified in paediatric dental traumatology? An exploratory study

**DOI:** 10.1186/s12903-023-03013-y

**Published:** 2023-05-10

**Authors:** Gertrude Van Gorp, Arno Maes, Marjan Lambrechts, Reinhilde Jacobs, Dominique Declerck

**Affiliations:** 1 KU Leuven Department of Oral Health Sciences and Department of Dentistry, Unit of Paediatric Dentistry and Special Dental Care, University Hospitals Leuven, Kapucijnenvoer 7, Leuven, PO box 7001, B-3000 Belgium; 2 KU Leuven Department of Oral Health Sciences, Master in Dentistry, University Hospitals Leuven, Leuven, Belgium; 3 OMFS IMPATH Research Group, Department of Imaging and Pathology, Faculty of Medicine, University of Leuven and Oral & Maxillofacial Surgery, University Hospitals Leuven, Leuven, Belgium; 4 Department Dental Medicine, Karolinska Institutet, Stockholm, Sweden

**Keywords:** Traumatic dental injury, Intra-oral radiographs (2D), Cone-beam CT (3D), Paediatric dentist, Radiographical diagnostic performance

## Abstract

**Background:**

Proper skills in radiographic diagnosis are essential for optimal management of dental trauma.

**Aim:**

To assess diagnostic accuracy obtained by paediatric dentists using Cone Beam Computed Tomography (CBCT) without specific training and to compare this with their performance using intraoral radiographs.

**Methods:**

Intraoral and CBCT images of 89 teeth, spread over twenty dental trauma cases were presented in random order to nine paediatric dentists. Diagnostic findings were compared with those of a benchmark reference. Sensitivity and specificity were calculated and compared using paired t-tests.

**Results:**

Overall, observers’ diagnostic performance was rather poor with significantly higher sensitivity when using 2D images (*P* = 0.017). Performance differed considerably according to the type of pathology. Using either imaging modality, sensitivity for diagnosing apical pathology and root fractures was high while the opposite was seen for inflammatory root resorption, root cracks and subluxations. Statistically significant differences between imaging modalities were seen for root fractures (*P* = 0.013) and apical pathology (*P* = 0.001), in favor of 3D, and for crown fractures (*P* = 0.009) in favor of 2D.

**Conclusion:**

Overall poor performance of paediatric dentists indicates that additional training in radiographic diagnosis is required. In order to justify the use of CBCT to increase diagnostic performance, proper training of the paediatric dentist is mandatory.

## Background

The evaluation, diagnosis and management of a traumatized dentition, affecting approximately 20% of children and adolescents, presents a challenge to clinicians [1, 2]. An accurate diagnosis guides the management of a traumatic dental injury (TDI) and influences the prognosis of the traumatized tooth [3]. Radiological examination, in addition to a detailed trauma history and a proper clinical examination, is an essential part of this process [2]. Diagnostic radiographs establish baseline records at the time of the initial examination and allow objective assessment at follow-up appointments [2]. The recommended radiographic standard for evaluating TDIs consists of intraoral radiographs (periapical and occlusal radiographs) taken from different angles [4]. Since Cone Beam Computed Tomography (CBCT) has become a widely available 3D technology in oral and maxillofacial radiology, this imaging modality is frequently applied to assist in clinical diagnosis of hard tissue pathology, also in young patients.

However, the use of CBCT in paediatric dental patients, a population with high prevalence of TDIs, must be carefully considered because the dose of ionizing radiation exceeds the dose used in conventional periapical radiography by a factor 20 to 400 [5]. Children’s higher radiosensitivity makes them more susceptible to radiation-induced malignancies [6]. Therefore, the use of CBCT scans in children can only be justified as a diagnostic method when intraoral radiography and clinical examination alone are unable to provide sufficient information [7]. In addition, proper training of clinicians for a safe and judicious use of CBCT in the dentoalveolar region is utmost important in a paediatric population, certainly with the readily availability of CBCT scanners in a growing number of private practices [8]. The decision to apply the third dimension for imaging purposes in a paediatric population must be taken with due responsibility, ensuring minimal risks and optimal benefits for the patient. Yet, the diagnostic performance of paediatric dentists, often primary care providers for dental trauma in young permanent teeth, has hardly been investigated [9]. Therefore, the present study explored this aspect in order to evaluate whether there is a need for (additional) training or stricter regulations regarding prescribing and interpretation of CBCT images.

The aim of this study was to assess the diagnostic accuracy for various pathologies after dental trauma obtained by paediatric dentists without specific training in the use of CBCT, and to compare this with their performance using intraoral radiographs (2D).

## Methods

### Study participants

All members of the Belgian Academy of Paediatric Dentistry (BAPD), 70 paediatric dentists in total in 2016, were invited by e-mail to participate on a voluntary basis in this research project. From sixteen paediatric dentists enrolled in the study, three participants were excluded because of incomplete data collection (no participation in one of both sessions, multiple missing answers). Scores of four observers were removed because they were considered as outliers based on predefined parameters (see below), either for 2D or for 3D images. Data obtained from the remaining nine observers was included in the analyses. Personal and professional information was collected using questionnaires and consisted of information about gender, postgraduate training and practice of paediatric dentistry.

### Selection of cases

Clinical and radiological material for this study was selected from a dental trauma database consisting of 502 cases with full clinical and radiological documentation, all of them treated by a single paediatric dentist between July 2010 and October 2016. Fifty-nine records (12%) containing both 2D intraoral radiographs (occlusal or periapical images) and 3D CBCT scans, prescribed in case of a complex dento-alveolar trauma, were available. From these records, 20 cases (34%) involving 89 teeth were selected based on following criteria: (1) radiographs were taken within a period of four months after the traumatic event; (2) with a maximum interval of three weeks between two- and three-dimensional imaging; (3) children were, at the moment of trauma, younger than 18 years and (4) cases presented a wide variety of TDIs in the anterior region. Case selection was based on the inclusion of 2D and 3D images that allowed expert diagnosis by both an experienced and trained endodontist and dentomaxillofacial radiologist. Cases with unacceptable diagnostic image quality caused by motion and/or metal artefacts were excluded from the present study.

### Image acquisition

Periapical radiographs were obtained using the paralleling long cone technique with 3 × 4 cm phosphor plates (VistaScan® image plate, Dürr Dental AG, Bietigheim-Bissingen, Germany). Occlusal radiographs were taken with a 7.5 × 7.5 cm phosphor plate (VistaScan® image plate, Dürr Dental AG, Bietigheim-Bissingen, Germany). The image receptors used to scan both types of phosphor plates were Digora Optime UV System (Soredex, Tuusula, Finland) or Vistascan mini plus (Dürr Dental AG, Bietigheim-Bissingen, Germany).

CBCTs were captured at the Dentomaxillofacial Imaging Center of the University Hospitals of Leuven with a 3D Accuitomo 170® CBCT (Morita, Kyoto, Japan) with small (6 × 6 cm) or medium (8 × 8 cm) field of view for respectively a voxel size of 0.125 mm or 0.160 mm.

### Case presentation and image evaluation

The paediatric dentists were invited to attend two separate sessions, both supervised by two moderators and organized in the same setting with 11 weeks in between. In this way viewer fatigue and the chance of recalling previous evaluations were minimized. At the beginning of the first session, eight training and calibration cases, not included in the main study, were presented to the participants in order to allow familiarization with the procedure.

All information of each of the 20 TDI cases was pseudonymized. A summary of the dental trauma history, supplemented with clinical pictures, relevant medical information, patient age and gender, clinical signs and symptoms (response to percussion and sensibility tests, probing depth, gingival bleeding, swelling, tooth mobility, pain) and emergency management was provided. Information and intra-oral images were incorporated in a PowerPoint presentation (PowerPoint 2013—Microsoft Office 2013) and displayed in optimal conditions without possibility to edit the digital images. To ensure optimal visualization of the CBCT dataset, observers were able to scroll and enhance original images by manipulation of the brightness and contrast using the viewing system’s software OneVolumeViewer® (provided by the manufacturer Morita, Kyoto, Japan).

Participants were installed in a dimmed room and radiological images were viewed on a 23 inch LED display monitor (Dell Ultrasharp U2312HM; Dell Corporation Ltd, Bracknell, UK) with resolution of 1920 X 1080, maximum brightness of 352 cd m^−2^ and dynamic contrast ratio of 10.000:1.

For either imaging modality, images were presented in random order and assessed independently by the observers. The observation time was ten minutes for 3D and three minutes for 2D images. Time allotted was based on a small pilot study carried out in paediatric dentistry trainees measuring the time required for observation and interpretation of radiological images. They are a reflection of the time needed in a clinical situation.

The radiological evaluation of 2D and 3D images serving as benchmark was performed by two specialists, a highly trained expert in Dento-maxillofacial Radiology (university professor) and a paediatric dentist experienced in the field of dental traumatology and endodontology. In case of disagreement, individual scores were discussed until consensus was reached.

### Reporting design

At the start of the first session, observers were instructed how to assess and how to report, for each individual case, all trauma-related injuries and pathoses on teeth visible on the radiographic images, using a pictorial reporting sheet for both 2D and 3D radiographs. The first part consisted of drawing each detected finding for 2D radiographs on an axial diagrammatic representation and for 3D radiographs on axial, sagittal and transversal diagrammatic representations. Following this, a description of each of the radiological findings was asked and finally the observers formulated a diagnosis based on radiological findings and clinical history.

### Data analysis

All data were collected and entered into an Excel file (Excel 2013—Microsoft Office 2013 Belgium, Dutch). A multivariate space spanned by three predefined quality parameters (number of false positives, number of false negatives and number of wrong identifications) was set up for 2D and 3D images separately. Outliers were defined as showing more mistakes than the median number of mistakes both on 2D and on 3D, when data analysis revealed a Mahalanobis distance, based on a robust estimation of covariance and location, larger than the 99.9th percentile of a chi-square distribution with 3 degrees of freedom. The Mahalanobis distance is a multivariate standardized distance that calculates the distance between an observation and the multivariate center of a certain data series, and corrects for the observed variability and correlation.

For each observer a list of diagnoses based on periapical radiographs and on CBCT scans, including acceptable variations in formulation, was compiled and compared with benchmark diagnoses. The number of correct, incorrect (missed, wrong) and overscored diagnoses was calculated. Observers’ radiographical diagnostic performance using either radiographic technique, overall and separately for various pathologies, was determined using the calculation of sensitivity and specificity. Differences in performance using 2D and 3D imaging were analysed using a paired t-test with significance level set at 5% (*P* < 0.05).

## Results

Scores obtained by four observers, who completed both sessions, were excluded from further analyses. Due to an answering pattern that differed considerably from that of the other observers, these four observers were considered as statistical outliers (see above). Differences in background education and training were not indicative of their performance. Data of nine paediatric dentists was included in the study. This group consisted of six females and three males with professional experience varying from less than five to up to 40 years and without specific training in CBCT. A new dental trauma case was seen from once a week to once every three months and complex trauma cases were frequently referred for specialized and multidisciplinary management.

The 20 included dental trauma cases yielded a total number of 89 permanent teeth to be evaluated. The mean age at the moment of trauma was 8.8 years (± 2.4), with a range from 5 to 15 years.

As shown in Table 1, the overall diagnostic performance was rather poor, using either imaging technique, with sensitivity scores ranging between 36.2% and 68.1% when using 2D images and between 30.1% and 51.6% when using 3D images. Differences in performance reached statistical significance favoring 2D imaging (*P* = 0.017). All observers, except observer 6, performed better using 2D. Specificity was high for both imaging modalities (mean and standard deviation 87.2% (± 4.3) using 2D and 89.2% (± 9.1) using 3D), without reaching statistical significance for the difference between both imaging modalities (*P* = 0.855).

**Table 1:** Overall diagnostic performance, expressed using sensitivity and specificity, using either 2D or 3D images (mean xpercentage and standard deviation)

	**Sensitivity**		**Specificity**	
	**2D**	**3D**	**2D**	**3D**
OBS 1	50.7	38.7	91.0	96.0
OBS 2	44.9	41.9	84.6	69.3
OBS 3	46.4	41.9	88.5	89.3
OBS 4	43.5	39.8	88.5	93.3
OBS 5	68.1	51.6	78.2	80.0
OBS 6	36.2	44.1	87.2	97.3
OBS 7	49.3	30.1	92.3	89.3
OBS 8	49.3	36.6	91.0	95.9
OBS 9	53.6	43.0	84.6	91.9
Mean (± SD)	49.1 (± 8.7)	40.9 (± 5.8)	87.2 (± 4.3)	89.2 (± 9.1)
*P*-value	0.017*		0.855	

*Abbreviations*: *OBS* Observer, *SD* Standard deviation

^*^Statistically significant difference *(P* < *0.05)*

Table 2 illustrates considerable variation in observers’ performance for diagnosing different trauma-related pathologies, using either 2D or 3D images. High sensitivity was seen for diagnosing root fractures and apical pathology, with statistically significant higher scores when using 3D images (*P* = 0.013, *P* = 0.001 respectively). Sensitivity for diagnosing crown fractures was higher using 2D images (*P* = 0.009). Low sensitivity, on 2D images as well as on 3D images, was noted for diagnosing subluxations (34.7% and 29.6% respectively), root cracks (22.2% and 14.8% respectively) and inflammatory root resorptions (1.4% and 6.2% respectively), without statistically significant differences between imaging modalities. None of the alveolar bone cracks was visible on 2D images; using 3D imaging, sensitivity was low (20.8%).

**Table 2:** Diagnostic performance, expressed using sensitivity and specificity, for different trauma-related pathologies using either 2D or 3D images (mean percentage and standard deviation)

	**Sensitivity**		**Specificity**	
	**2D**	**3D**	**2D**	**3D**
**Crown fracture** *P*-value	61.7 (± 19.8)	39.8 (± 25.9)	100.0 (± 0.0)	99.8 (± 0.7)
	0.009*		0.346	
**Root fracture** *P*-value	85.7 (± 0.0)	94.4 (± 8.3)	99.2 (± 1.0)	100.0 (± 0.0)
	0.013*		0.035*	
**Root crack** *P*-value	22.2 (± 36.3)	14.8 (± 12.4)	98.3 (± 1.9)	98.9 (± 2.1)
	0.522		0.783	
**Alveolar bone crack**	/	20.8 (± 15.3)	/	98.1 (± 5.2)
**Subluxation** *P*-value	34.7 (± 23.2)	29.6 (± 18.9)	94.7 (± 4.1)	97.6 (± 3.5)
	0.447		0.002*	
**Apical pathology** *P*-value	60.2 (± 8.1)	80.7 (± 13.1)	98.2 (± 1.3)	98.1 (± 1.7)
	0.001*		0.852	
**Inflammatory root resorption** *P*-value	1.4 (± 4.2)	6.2 (± 11.3)	100.0 (± 0.0)	98.8 (± 1.8)
	0.099		0.095	

/ = no observations

^*^ Statistically significant difference *(P* < *0.05)*

Inflammatory root resorption was the most frequently missed finding, both on 2D and 3D (72.2% and 58.0% of cases respectively). This was also the most frequently incorrectly diagnosed finding (26.4% on 2D, 35.8% on 3D). Also subluxation was frequently missed (55.5% on 2D, 61.1% on 3D) or incorrectly diagnosed as apical pathology (6.3% on 2D, 5.6% on 3D). Presence of apical pathology was missed in 37.0% of cases on 2D and 17.0% on 3D. This pathology was incorrectly diagnosed as subluxation (2.8% on 2D, 2.2% on 3D) or diagnosed when not present (8.3% on 2D, 6.7% on 3D).

**Fig. 1:**
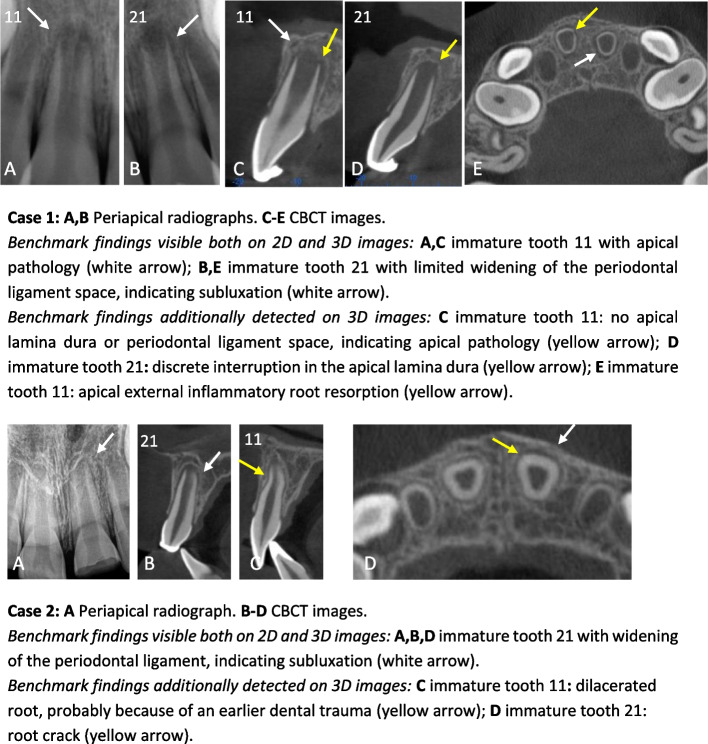
Two examples of clinical cases illustrating the benchmark diagnoses on 2D and on 3D images. Case 1: **A**, **B** Periapical radiographs. **C**-**E** CBCT images. *Benchmark findings visible both on 2D and 3D images: ***A**, **C** immature tooth 11 with apical pathology (white arrow); **B**, **E** immature tooth 21 with limited widening of the periodontal ligament space, indicating subluxation (white arrow). *Benchmark findings additionally detected on 3D images: ***C** immature tooth 11: no apical lamina dura or periodontal ligament space, indicating apical pathology (yellow arrow); **D** immature tooth 21: discrete interruption in the apical lamina dura (yellow arrow); **E** immature tooth 11: apical external inflammatory root resorption (yellow arrow). Case 2: **A** Periapical radiograph. **B-D** CBCT images. *Benchmark findings visible both on 2D and 3D images: ***A**, **B**, **D** immature tooth 21 with widening of the periodontal ligament, indicating subluxation (white arrow). *Benchmark findings additionally detected on 3D images: ***C** immature tooth 11**:** dilacerated root, probably because of an earlier dental trauma (yellow arrow); **D** immature tooth 21: root crack (yellow arrow)

Examples of clinical cases used in this research, illustrating benchmark diagnoses, are presented in Fig. 1. In case 1, the apical lesion present on tooth 11 was missed by 2 observers on 2D and by 4 observers on 3D; apical pathology was scored on tooth 21 by 4 observers on 2D and by 1 observer on 3D, although not present. The external inflammatory root resorption was correctly diagnosed by only 1 observer on 3D. In case 2, the subluxation on tooth 21 was missed on 2D by 5 observers and on 3D by 3 observers; subluxation was misdiagnosed as apical pathology by 2 observers, both on 2D and on 3D; the root crack in tooth 21, visible on 3D, was missed by 7 out of 9 observers.

## Discussion

Detailed clinical and radiological evaluations are essential for diagnosing, assessing and following-up traumatic dental injuries. The ability to accurately interpret a radiographic image is essential for obtaining a correct diagnosis of dentoalveolar abnormalities, mandatory to optimal patient management, and to avoid unnecessary and inappropriate treatment [2].

This study evaluated paediatric dentists’ diagnostic ability of dental trauma lesions using different imaging modalities: intraoral radiographic techniques (periapical and occlusal) or CBCT. The paediatric dentists who participated in this study had little to no previous training in using CBCT.

Overall, the performance of the observers was rather poor, using either imaging technique, indicating a clear need for additional training. This confirms earlier reports indicating a need for continuing education in conventional intraoral radiography [10, 11]. With the increased availability and use of CBCT, this also applies to imaging using the third dimension [8, 12]. The latter is particularly important since it will not only contribute to minimizing unnecessary radiation exposure without yielding added value, but also to improving the diagnostic process of dental trauma lesions with impact on their management and the prognosis of affected teeth [8, 12]. The overall better performance of the observers when using 2D images can be explained by the fact that they received more training in the use of this technique, mainly as part of undergraduate training. They are much more familiar with the use of 2D and more competent in diagnosing pathologies applying this imaging modality [13].

Interestingly, this study demonstrated a wide variability in diagnostic performance according to different types of TDI related pathologies. Well-recognized pathologies were root fractures; defined as fractures involving damage to pulp, dentin, cementum and periodontal ligament [14], and apical pathology, with 3D imaging yielding significantly superior results. These findings are in line with earlier reports concluding that CBCT should be regarded as an accurate and reliable imaging modality for a more precise evaluation of the location, extension and direction of root fractures [15, 16].

Also the assessment of the presence of apical pathology has been reported to be facilitated by using the third dimension [16, 17]. Overlooking a diagnosis of apical pathology will result in disease progression and delay in endodontic management, compromising the prognosis of the affected tooth. This illustrates the added value of imaging using the third dimension in these situations.

A different picture was seen regarding the radiographic diagnosis of crown fractures, with better performance using 2D imaging. This observation may be explained by artefacts affecting CBCT image datasets much more than it is the case for intra-oral radiographs [18]. Such artefacts may be caused by high dense materials, such as ceramic restoration materials, sealers, yet also by enamel in the region of the crown but not the root.^18^ Such artifact expression may further increase by motion [19].

Using either imaging modality, paediatric dentists showed poor performance regarding the diagnosis of subluxation, inflammatory root resorption and root cracks. Subluxations were not only frequently overlooked, but also often misdiagnosed. Diagnosing the widening of a periodontal ligament space as presence of apical pathology when in fact subluxation is present, will lead to unnecessary invasive interventions such as endodontic treatment of the affected tooth [20]. In order to reduce diagnostic confusion in a situation of widened periapical ligament space, careful and detailed radiological interpretation of the periapical region remains essential [21, 22].

Inflammatory root resorption was the most frequently missed and underdiagnosed pathology, using either imaging modality. Inflammatory root resorption is linked to pulp necrosis and the resorptive defects appear as periradicular radiolucencies on the root surface [23]. Early radiographic diagnosis of inflammatory root resorption and adequate endodontic treatment are essential to manage this condition [24]. The superior diagnostic performance obtained when using CBCT for the detection of resorptive lesions, compared with intraoral radiographs, has been reported in the literature, [25, 26] but could not be confirmed in the present study. The poor diagnostic performance of the observers regarding this condition and the diversity in terminology used to describe these lesions (apical root resorption, internal / external root resorption, cervical root resorption) are indicative of an important knowledge gap. The correct diagnosis of root resorptions is indeed challenging, with inadequate assessment resulting in inappropriate treatment approaches [23].

Root cracks or incomplete root fractures were often missed, using either technique. As reported in the literature, an incomplete or partial root fracture on an immature tooth defined as a unilateral break in the continuity of the thin root wall, will heal with hard tissue formation and without any endodontic intervention [27]. This means that the clinical impact of missing or overlooking root cracks is rather low.

This study showed that the use of CBCT without specific training did not improve the overall diagnostic performance of paediatric dentists in case of TDIs, despite the fact that CBCT is becoming a commonly used diagnostic imaging tool in dentistry. This is not in accordance with reports describing that CBCT provided superior interpretation of information with a better insight into diagnostic dilemmas and complex treatment decisions [28–31]. However, it should be emphasized that in these studies observers were specialists in endodontics or in oral maxillofacial radiology, all of them better trained in CBCT reading. This underlines the need for incorporating more extensive training in CBCT interpretation in the curriculum of dental practitioners. If not adequately trained, referral to an oral and maxillofacial radiologist should be considered in case of complex TDIs [29].

Surprising was the rather poor performance of paediatric dentists when interpreting conventional intraoral radiographs, suggesting the need for more training also in these aspects of oral radiology.

It is fair to discuss some limitations of the study since some of these aspects could have possible impact on the validity and generalisability of the present study. First, the set-up used for this research does not represent the daily routine of a dental practice. Indeed, the examiners were not able to manipulate the intra-oral radiographs or to consider clinical examination of the patient since all information and intra-oral radiographs were presented incorporated in a PowerPoint presentation. In addition, the ten minutes provided for CBCT reviewing might not have been enough for a non-experienced user to fully benefit from exploring the 3D scans. Furthermore, a higher number of observers as well as including observers with different training background and levels of experience in CBCT could provide a broader and more detailed picture allowing broader extrapolation of the results. Also, a higher number of clinical cases with even broader variety of clinical situations could be envisaged. However, based on the information obtained in this exploratory study, well-documented sample size calculations can be undertaken for setting up larger studies exploring this topic.

Also the justification for the use of CBCT in the selected cases should be discussed. Images were collected from a database of patients treated in the time period between July 2010 and October 2016. Given the complexity of the dentoalveolar trauma cases, the indication for obtaining a CBCT was considered on a case by case basis. Recently, best clinical practice guidance recommendations were published by the European Academy of Paediatric Dentistry, indicating insufficient evidence to recommend the standardized use of CBCT for an acute dental trauma or in cases of late complications after dental trauma [7].

This research, the first study exploring paediatric dentists’ diagnostic accuracy of various dental trauma related pathologies, indicates the presence of important knowledge gaps using either intraoral radiographs or CBCT images with a clear need for enhanced training in radiographic diagnosis. In case of complex TDIs, referral to a professional with specific expertise in radiographic diagnosis should be envisaged. The use of CBCT without proper training in CBCT imaging and related diagnostic interpretation is not justified.

Based on the results of this research, a larger study involving more observers with different levels of experience and training seems to be indicated. Also, further exploration of the impact of incorrect (missed, wrong) and overscored diagnoses on performed treatment could be of interest. Further research is also needed to unravel the cost benefit aspect of 2D versus 3D diagnosis in the field of oral paediatric traumatology. This should not only consider costs involved with diagnostic acts but also benefits obtained from improved diagnosis, enhanced therapeutic approaches and better prognosis.

## Data Availability

The datasets generated and/or analyzed during the current study are not publicly available due to privacy policies of the University Hospitals Leuven but are available from the corresponding author on reasonable request.

## Abbreviations

TDI: Traumatic Dental Injuries
CBCT: Cone Beam Computed Tomography
